# Oral extrusion of a vertebral body replacement device after chordoma tumor growth and radiation: case report and review

**DOI:** 10.1186/s12893-022-01481-7

**Published:** 2022-01-22

**Authors:** Raquel Gutiérrez-González, Álvaro Zamarrón, Celia Ortega, Frank Hamre, Teresa Kalantari, Gregorio Rodríguez-Boto

**Affiliations:** 1 Department of Neurosurgery, “Puerta de Hierro” University Hospital, C/. Joaquin Rodrigo 2, 28222 Majadahonda, Madrid Spain; 2 Department of Surgery, Faculty of Medicine. Autonomous, University of Madrid, C/. Arzobispo Morcillo 4, 28019 Madrid, Spain

**Keywords:** Case report, Cervical, Device, Failure, Pharyngeal perforation

## Abstract

**Background:**

Screw migration following anterior cervical discectomy and fusion is a very rare complication and it is often related to device failure. Even more exceptional is the extrusion of an intervertebral graft.

**Case presentation:**

We report the second case of migration and extrusion through the oral cavity of a cervical vertebral body replacement device (expandable cylinder) in a patient that had undergone cervical corpectomy due to a vertebral chordoma.

**Conclusion:**

The antecedent of radiation therapy as well as progressive tumor re-growth may have favored the development of this complication. A literature review is added.

## Background

Screw migration following anterior cervical discectomy and fusion is a very rare complication and is often related to device failure. It may provoke pharyngeal or esophageal perforation and, eventually, the inadvertent and spontaneous expulsion of the screw through the gastrointestinal tract [2, 8, 17, 21, 23]. Oral expulsion of the screw has been occasionally reported [5, 7, 9, 20], as well as the exposure or extrusion of the plate going with the screw [6, 16, 26]. Even more exceptional is the extrusion of an intervertebral graft following anterior cervical discectomy and fusion [1, 10, 11, 15, 18, 19, 22]. Despite the sometimes uneventful course of the complication, the importance lies on the risk of fistula and infection that usually entail slow recovery.

In this article, we report the exceptional case of a patient diagnosed with a cervical chordoma that presented at clinics with sudden extrusion of a cervical vertebral body expandable cylinder through the oral cavity.

## Case presentation

**Fig. 1:**
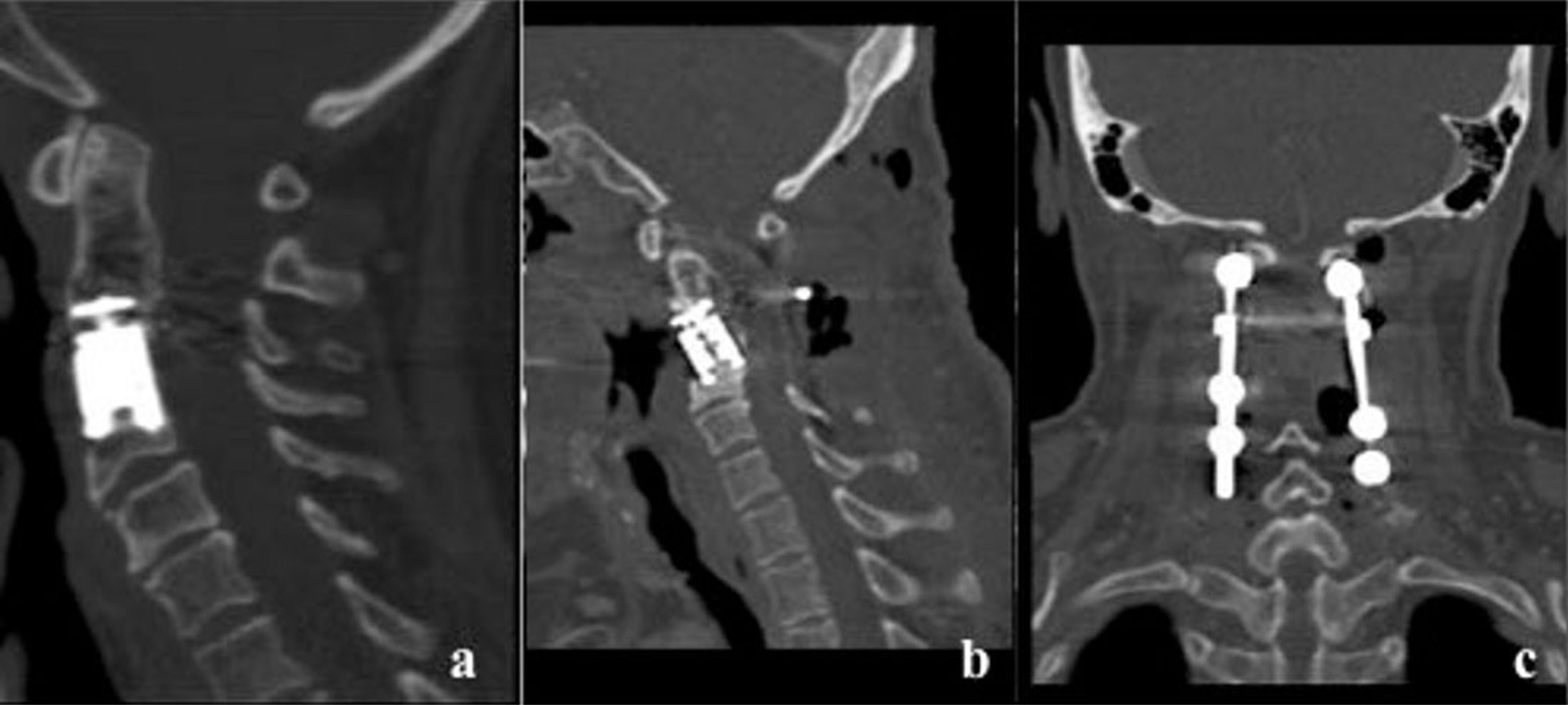
**a** Computed tomography (CT) image after the first surgery shows well-aligned C3 interbody expandable device; **b***, ***c** CT images after the last surgical procedure show partial interbody device failure and posterior cervical stabilization

A 59-year-old Caucasian female was operated on in 2005 when she was diagnosed with a cervical tumor invading the vertebral body of C3. She underwent an anterior transmandibular approach achieving tumor resection and anterior stabilization with an interbody expandable cylinder device (Fig. 1a). Histological analysis evidenced a chordoma and the patient underwent subsequent intensity-modulated radiotherapy (50 Gy). Six years later she presented with tetraparesis that was related to tumor relapse. Again, she underwent a left anterolateral submandibular approach to achieve tumor resection and spinal cord decompression. A minimal piece of tumor remained adhered to the vertebral artery. Neurological recovery was complete.

**Fig. 2:**
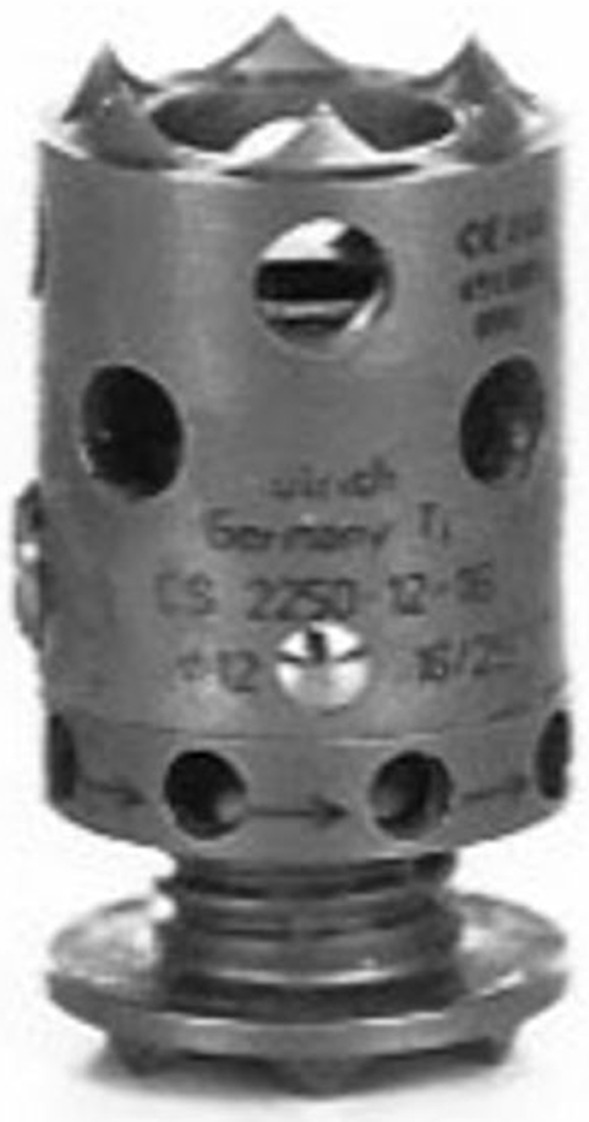
Interbody expandable device as shown by the patient at clinics

**Fig. 3:**
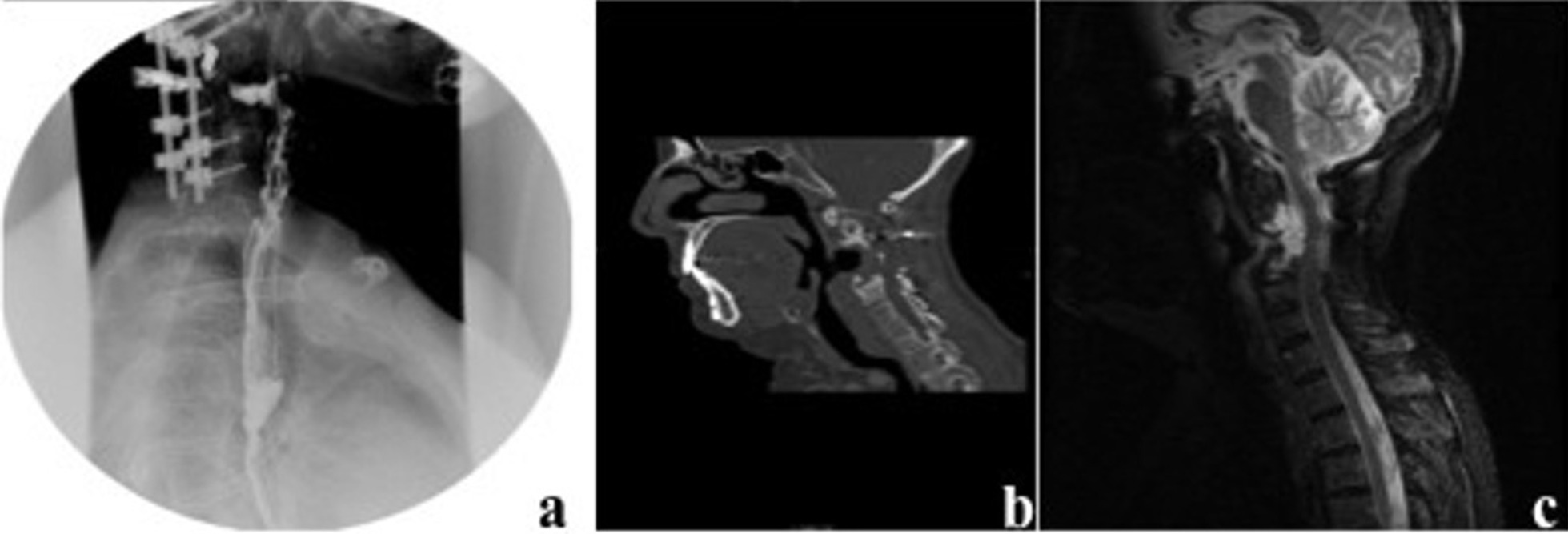
**a** Esophagram test and **b** CT scan show the decubitus ulcer in the posterior wall of the oropharynx; **c** magnetic resonance image shows tumor relapse

**Fig. 4:**
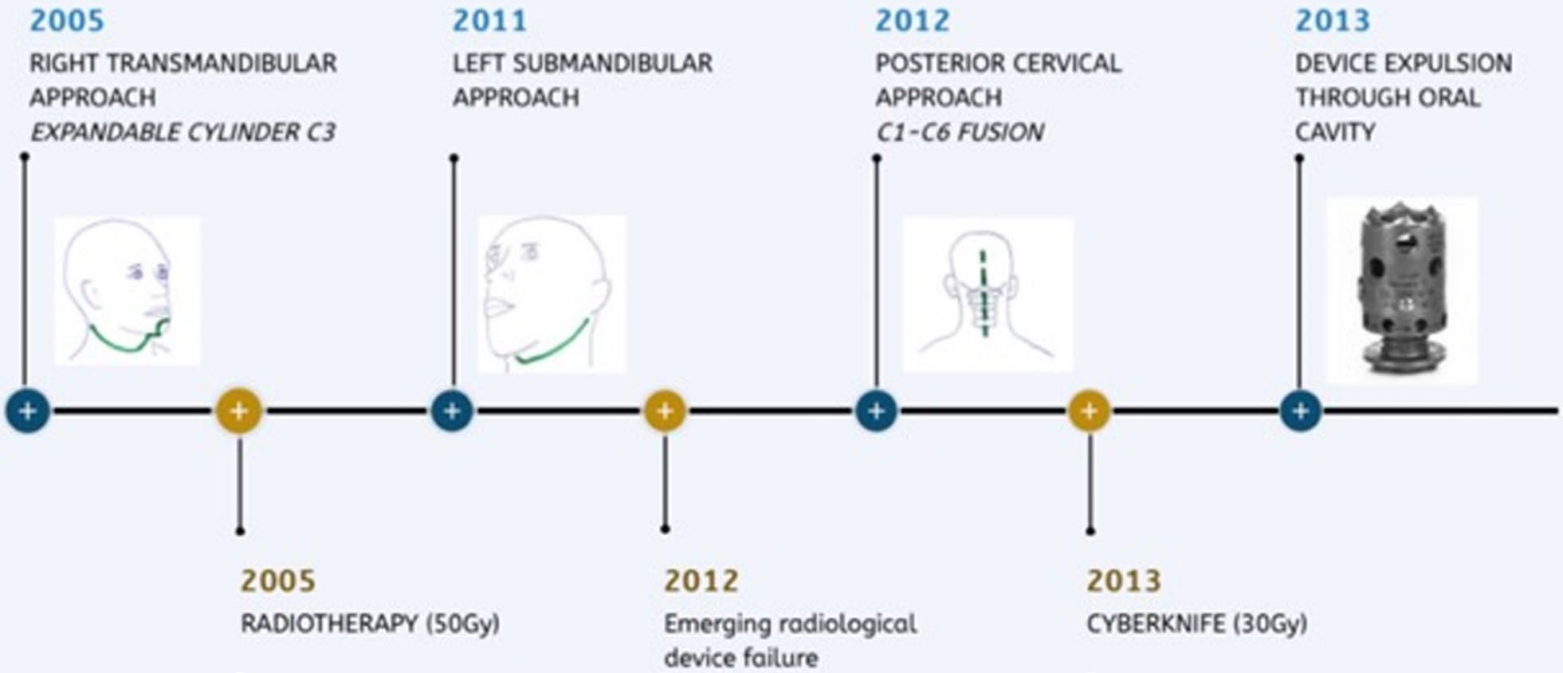
Scheme on the surgical and therapeutic method followed

One year later the patient showed a new recurrence that invaded the vertebral bodies of C2 and C3. At this point a posterior approach was accomplished to resect the tumor and add spine stabilization C1–C6 (Fig. 1b and c). Following the procedure, the patient underwent adjuvant therapy with Cyberknife (re-irradiation with 30 Gy). Once the treatment had finished the patient noted progressive halitosis and dysphagia. She was attended at our center after sudden extrusion of the titanium cylinder that had been implanted during the first surgical operation. She referred a cough access during deglutition that resulted in the device expulsion through the oral cavity (Fig. 2). Fiber laryngoscope evaluation showed left hypopharynx widening and ipsilateral piriform recess collapse. The radiological studies showed a fistulous tract related to a decubitus ulcer in the posterior wall of the oropharynx as well as the absence of the interbody device in C3 (Fig. 3). The patient recovered uneventfully after conservative management and endovenous antibiotic therapy. Four years later, she is waiting for a new surgical procedure due to tumor progression. Figure 4 summarizes the surgical and therapeutic methods that the patient underwent throughout the process.

## Discussion

Oral extrusion of spinal stabilization devices is a very rare complication. It may be due to pharyngeal or esophageal perforation. It has been described regardless of the material and type of graft implanted [1, 4, 10, 11, 15, 18, 19, 22]. Thus, as far as six cases of oral extrusion of a cervical disc replacement device have been reported to date [1, 10, 15, 18, 19, 22]. Another case of oral extrusion of an anterior bone graft implanted after tissue debridement due to infection has been included in this review, despite scarce data [11]. However, there is only one case of vertebral body replacement device extrusion published up to date [4]. The case hereby reported is therefore the second one, and both of them share similar features, such as the oncological underlying process, the delayed course, the presence of a posterior arthrodesis besides the anterior one, or the adjuvant treatment with radiotherapy. Table 1 summarizes the specific features of these patients.

**Table 1:** Reported cases of oral extrusion of stabilization devices of the cervical spine

Author	Age/Sex	Ethiology	Symptoms	Device	Delay	Results
Louw [11]	28/M	C2C6 debridement	Lump, vomit	Iliac bone graft	6 wk	Resolution
Ogle et al. [15]	36/F	ACDF (degenerative)	Dysphonia + dysphagia, cough	Iliac bone graft	5 yr	Resolution
Cavanagh et al. [1]	74/M	C4C5 ACDF (degenerative)	Cough	BOP graft	14 wk	Resolution
Sharma et al. [22]	33/F	C2C3 ACDF (dislocation)	Pain, dysphagia, cough	Iliac bone graft + pin	1 year	Resolution
Lin et al. [10]	45/M	C4C5 ACDF (degenerative)	Cough	BOP graft	4 yr	Resolution
Faguer et al. [4]	19/F	C4 Ewing’s sarcoma	Spontaneous extrusion	PMMA graft + kirschner’s pin	12 yr	Resolution
Quadri et al. [19]	84/F	C2C3 ACDF (fracture)	Pain, cough	Plate + screws + PEEK graft	3.5 yr	Lost follow-up
Prusick et al. [18]	51/F	C2C3 ACDF (degenerative)	Dysphagia, cough	Integrated plate-cage + screw	1.5 yr	Resolution
Present case	66/F	C3 chordoma	Halitosis, dysphagia, cough	Vertebral body expandable cylinder	7 yr	Resolution

*ACDF* anterior cervical discectomy and fusion, *BOP* biocompatible osteoconductive polymer; *PEEK* polyetheretherketone; *wk* weeks; *yr* years

Most of the cases presented with any kind of symptom before a cough access that was responsible for device extrusion in all cases except one, in which the extrusion was spontaneous during deglutition [4]. Three patients showed progressive radiological device failure in follow-up studies prior to the extrusion [4, 10, 22] as well as the present case. However, since a posterior fusion had also been performed, it was considered enough to stabilize the spine.

The incidence of instrumentation failure following anterior cervical plate fixations is believed to be higher than expected (18%), but only 7% of them may need surgery to fix it [12]. However, a recent systematic review of the literature lowers the complication value to 2.1% [25]. Another study registers an incidence of acute implant extrusion < 1% [24]. Tumor growth and preoperative irradiation, in addition to biomechanical forces, may have contributed to shift a vertebral body replacement device in the present case. Similarly, the case reported by Faguer et al. [4] would be explained both by prior radiotherapy together with the biomechanical forces derived from a growing spine in the pediatric age, in absence of tumor relapse [14]. Strategies to prevent such complication may include an anterior plate in addition to the cylinder or a circumferential fusion [24]. The anterior plate was discarded during the first procedure due to technical nuances (lordotic angle) and the posterior fusion was not performed at that moment in order to avoid the functional limitation at such an early stage of the disease. A posterior arthrodesis was considered during the second procedure, but it was finally discarded due to the functional status of the patient (tetraparesis). Prior right and left approaches could trigger the incipient device failure observed before the third surgical procedure, when the posterior fusion was finally added.

Besides that, radiation therapy is known to interfere in the process of bone fusion [3]. Radiation dose is also known to increase damage to the esophagus [13]. Thus, tumor growth and radiation therapy may have favored device failure in the case hereby reported, and device failure in addition to cervical re-irradiation may be responsible for esophageal erosion and perforation.

Pharyngeal or esophageal perforation must be accurately treated in order to avoid severe or even fatal infectious complications. Management includes antibiotics, device removal, repair of the defect, and nasogastric/gastrostomy/jejunostomy tubes in order to favor defect healing [12, 16, 23]. However, all cases included in this review evolved satisfactorily in absence of direct repair of the defect.

## Conclusions

We report the second case of migration and extrusion through the oral cavity of a cervical vertebral body replacement system after hypopharynx perforation in a patient that had undergone cervical corpectomy due to a vertebral chordoma. Neurological deficit was avoided thanks to the presence of a posterior cervical fusion. The antecedent of radiation therapy, as well as progressive tumor re-growth, may have favored the development of a decubitus ulcer in the pharynx and the migration of the interbody device respectively.

## Data Availability

All data generated or analyzed during this study are included in this published article.

## Abbreviation

Gy: Gray
